# First Coronavirus Active Survey in Rodents From the Canary Islands

**DOI:** 10.3389/fvets.2021.708079

**Published:** 2021-08-18

**Authors:** Abir Monastiri, Natalia Martín-Carrillo, Pilar Foronda, Elena Izquierdo-Rodríguez, Carles Feliu, Marc López-Roig, Jordi Miquel, Meriadeg Ar Gouilh, Jordi Serra-Cobo

**Affiliations:** ^1^Department of Evolutionary Biology, Ecology and Environmental Sciences, Faculty of Biology, University of Barcelona, Barcelona, Spain; ^2^Faculty of Biology, Institut de Recerca de la Biodiversitat, University of Barcelona, Barcelona, Spain; ^3^Instituto Universitario de Enfermedades Tropicales y Salud Pública de Canarias, Universidad de La Laguna, La Laguna, Spain; ^4^Department of Obstetricia y Ginecología, Pediatría, Medicina Preventiva y Salud Pública, Toxicología, Medicina Legal y Forense y Parasitología, Universidad de La Laguna, La Laguna, Spain; ^5^Department of Biology, Health and Environment, Faculty of Pharmacy, University of Barcelona, Barcelona, Spain; ^6^Normandie Université, EA2656, Groupe de Recherche sur l'Adaptation Microbienne, Caen, France; ^7^University Hospital of Caen, Virology Department, Caen, France

**Keywords:** *Betacoronavirus*, *Embecovirus*, *Murine coronavirus*, rodents, Canary Islands, coronavirus

## Abstract

Since the beginning of the 21st century five new coronaviruses inducing respiratory diseases in humans have been reported. These emergences has promoted research on coronaviruses in wildlife. We started the first eco-epidemiological study to screen the presence of coronaviruses circulating in mice and rats of four Canary Islands. Between 2015 and 2019, we obtained fecal samples of three rodent species (150 *Mus musculus*, 109 *Rattus rattus* and 1 *Rattus norvegicus*) captured in urban and rural areas. Fecal samples were analyzed by nRT-PCR and the resulting sequences were compared to known diversity using Bayesian phylogenetic methods. We only found coronavirus RNA in house mice from El Hierro (10.53%), Tenerife (7.02%) and Lanzarote (5.26%) islands. All coronaviruses detected belong to the species *Murine coronavirus* belonging to the genus *Betacoronavirus* and subgenus *Embecovirus*, being all positive house mice captured in anthropogenic environment. The phylogenetic analysis shows that murine coronaviruses from the Canary Islands are related to European murine coronaviruses. Albeit data are still scarce in the region, the most probable origin of *M. coronavirus* present in the Canary Islands is continental Europe. According to temporal Bayesian phylogenetics, the differentiation between Canary and continental viruses seems to be quite recent. Moreover, murine coronaviruses from El Hierro, Tenerife and Lanzarote islands tend to segregate in different clades. This enlightens the potential role of rodents or other possibly invasive species in disseminating infectious diseases to remote places through exchanges with the continent. It is important to consider these aspects in the sanitary control of islands, for health and biodiversity preservation concerns.

## Introduction

Although the first coronavirus (CoV) was isolated in chicken embryos in 1937 (1), it was not until the emergence of SARS-CoV in 2002-2003 that CoVs were considered major humans pathogens. Since the beginning of the 21st century five new coronaviruses inducing respiratory diseases in humans have been reported including SARS-CoV, HCoV-NL63, HCoV-HKU1, MERS-CoV, and SARS-CoV-2. This reminds the importance of coronaviruses in epidemic diseases. These viral emergences promoted research on wildlife viruses and light was shed on the extreme diversity of coronaviruses in nature (2–4).

Coronaviruses belong to the Coronaviridae family and are classified into four genera, *Alphacoronavirus, Betacoronavirus, Gammacoronavirus* and *Deltacoronavirus*. Within *Betacoronavirus*, they can be further subclassified into four subgenera *Embecovirus, Sarbecovirus, Merbecovirus* and *Nobecovirus* (5). Coronaviruses show respiratory and enteric tropism mainly, but may have hepatic and neurological tropism in animals and humans as well. Recombination has an evolutionary importance in coronaviruses and may promote the infection of new hosts and alter virulence (6).

Rodents are the largest order of mammals composed of more than 2,000 species, which represent more than 40% of all mammalian species. Rodents are a major zoonotic source of human infectious diseases, they often live at high densities and hence may harbor high levels of microbial diversity (7). On the other hand, some species are synanthropic and live in vicinity to humans so they may represent a zoonotic risk.

Mice and rats are not autochthonous species of the Canary Islands. They were introduced in the Archipelago long time ago and have spread widely throughout the islands. Findings suggest that the introduction of *Rattus rattus* in Lanzarote occurred before European contact at the Middle Age (8, 9), while *Rattus norvegicus* introduction is more recent, probably around the eighteenth century. On the other hand, the arrival of the house mouse *Mus musculus* occurred around 7,000 years ago (10, 11). Taking into account that in recent years a great diversity of alphacoronaviruses and betacoronaviruses have been identified in rodents in China and Europe (12–15), we started an eco-epidemiological study to screen the CoVs circulating in mice and rats of four Canary Islands. The present paper reports outcomes of the first results from this study.

## Materials and Methods

### Localities Description

The Canary Islands are of volcanic origin and they are located in the Atlantic Ocean, in North-Western Africa, belonging to the Macaronesia region. The climatology of the Canary Islands is subtropical being highly influenced by the North Trade winds. The average temperature and annual precipitation at the coast zone are 21**°**C and 160–266 mm, respectively, and 9**°**C and 500–800 mm at the highest altitudes (16). This contrast of weather conditions is driven by the orography, altitude and orientation of the islands, and have a great influence on the type of vegetation.

This Archipelago is divided into Eastern islands (Gran Canaria, Lanzarote and Fuerteventura) and Western islands (El Hierro, La Gomera, La Palma and Tenerife). The Eastern islands as well as the lowlands of the Western islands are characterized by a dry xerophytic environment with a scarce vegetation cover while the Western islands are more humid because of the effect of the horizontal rain, especially in the islands of Tenerife and La Palma. In these islands, the diversity of bioclimatic floors is highest than in the Eastern islands. Furthermore, Western islands display higher mountain areas, with the highest altitude located in Tenerife at 3,718 m (17).

We captured rodents in Frontera, a coastal area from the northern of El Hierro island formed by shrubs of low density. We sampled Barlovento, Breña Alta, Puntallana, San Andés and Los Sauces localities from La Palma island, and El Batán, La Laguna, Tegueste, El Sauzal, La Guancha and La Orotava localities from Tenerife island. These localities have high vegetation cover with high humidity. Furthermore, we sampled dry localities formed by shrub such as El Paso from La Palma, and Arona, Fasnia, San Miguel de Abona, Santa Cruz de Tenerife and Guía de Isora from Tenerife island. In the Eastern islands, we sampled in Femés, Haría and Maguez dry crop field localities from Lanzarote. Unlike the other islands included in this study, Lanzarote has low altitude that generates little contrast in vegetation.

### Sampling

**Figure 1:**
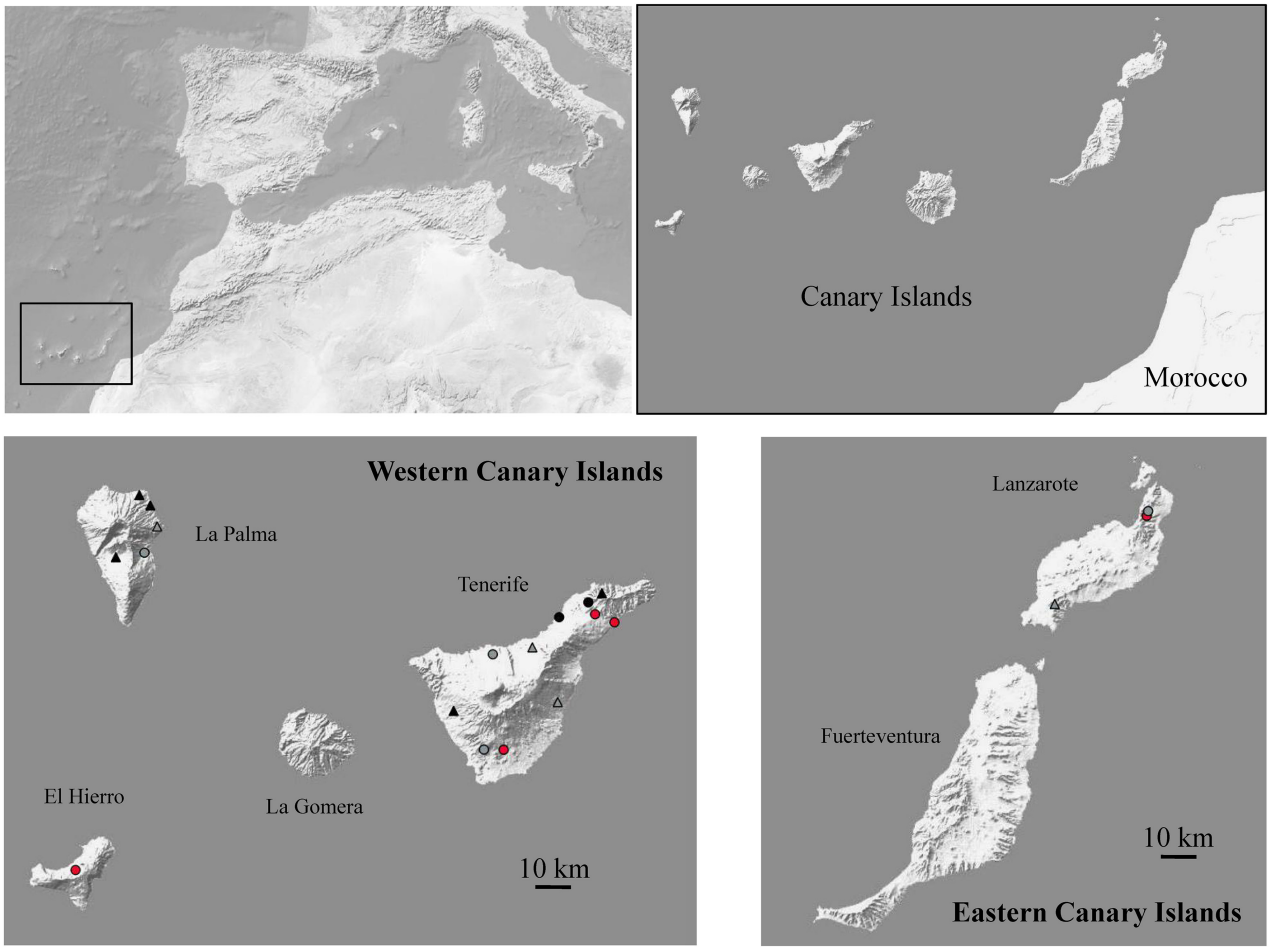
Map of the region studied showing the 20 investigated sites and highlighting in red those where samples were found positive for coronaviruses. Black and gray indicate localities where samples were found negative for coronaviruses with presence or absence of *Mus musculus*, respectively. Circles and triangles indicate anthropic and no anthropic localities, respectively.

Between 2015 and 2019, house rats (*R. rattus*), brown rats (*R. norvegicus*) and house mice (*Mus musculus*) were captured with Firobind-traps set in urban and rural areas (Figure 1). Traps with bait, composed of oil, flour and tuna, were set in the afternoon, with 5 meters of separation between them, and were collected the next morning. The traps were placed in rural and urban areas. Rural areas mainly included arid habitats, crop fields, private gardens and farms, whereas urban areas corresponded to highly populated zones. Rodents were morphologically identified considering that in the Canary Islands live only two *Rattus* species (*R. rattus* and *R. norvegicus*) and one mice species (*M. musuclus*). Once captured, rats were euthanized with CO_2_ and mice by cervical dislocation, and fecal samples were obtained and stored in RNA later (Invitrogen) until analysis. This study is a part of a multidisciplinary project that includes parasitological analysis (Pathogens and reservoirs in Atlantic and Mediterranean island ecosystems: interest in public health and the environment, Pro ID 2017010092). Animal procedures were conducted in accordance with the Government Laws of 42/2007, 151/2001 (expedient numbers A/EST-030/2016 and AFF115/16) and RD 630/2013 (expedient references EEI-007/2019, MRR/rsh and ADL/mjb).

### Sample Preparation and Detection of Coronavirus RNA

The fecal suspension was clarified at 5,000 g for 10 min at 4°C and total RNA was extracted from 150-μl supernatant using the Nucleospin Dx virus kit (Macherey-Nagel, Düren, Germany) following the manufacturer's instructions. The reverse transcription (RT) step was performed using the SuperScript Vilo cDNA Synthesis kit (Invitrogen, Carlsbad, CA, USA) following the manufacturer's instructions using 14 μl of extracted RNA in a 20 μl total reaction volume.

The first and the second PCR reactions (hemi-nested) were carried out using the Taq PCR Core kit (Qiagen, Hilden, Germany) following the manufacturer's instructions, using pan-coronavirus primer set (PanCoV pol 15197/PanCoV pol 15635/PanCoV pol nested 15419) previously described (4, 18). Five microliters of the RT product were used in first step PCR, in 50 μl final volume. For the second PCR reaction (hemi-nested), 5 μl of amplified product from the first PCR were added to 50 μl of the total reaction mixture containing 50 pmol of the second primer set (PanCoV pol 15635/PanCoV pol nested 15419). This methodology amplifies a conserved region of the ORF1.

After amplifications, 10 μl of the first PCR and the second (hemi-nested) PCR products were loaded into a 2% GelRed (Biotium) stained agarose electrophoresis gel in TBE buffer and visualized under ultraviolet light. Amplicon sizes (440 and 220 bp for first and second PCR, respectively) were determined by comparing them with a 100-bp DNA ladder (Qiagen, Barcelona, Spain). PCR products were purified using the PureLink PCR Purification kit (Invitrogen, Carlsbad, CA, USA) and bidirectionally sequenced (Macrogen Inc., Seoul, South Korea).

### Phylogenetic and Temporal Reconstruction Analyses

Quality trimming, assembling and consensus generation were performed using CLC main Workbench software (QIagen^©^). Alignment of consensus original sequences, with sequences representing the genetic diversity of coronaviruses were performed using MAFFT (19). PhyML, implemented in Seaview, was used to build the first maximum likelihood tree (20). The GTR+I+G model was used for the tree search and completed with best tree refinement using NNI and TBR. The whole maximum likelihood analysis was launched five times, the best tree was selected, and a likelihood ratio test was used to evaluate node support. The most appropriate model of evolution according to the corrected Akaike Information Criterion (AICc) was selected using JmodelTest2 (21) and Bayesian phylogenetic analyses were performed with BEAST (version 1.8.4) (22). The general time reversible model of substitution with a gamma distribution and a proportion of invariant sites (GTR+I+G) was used. The coalescent (constant size) model was used for building the random starting tree. Tip date sampling was combined with a relaxed molecular clock including uncorrelated lognormal distribution (23). The MCMC (Markov-Chain Monte Carlo) was launched with 5.10 E7 iterations and 10 E4 samples. Log and tree files were analyzed using tracer and fig tree, after a 10% discard to reach Effective Sampling Size (ESS) values above 200 and visualize the maximum clade credibility tree.

## Results

We analyzed 109 fecal samples of house rat (*R. rattus*), 1 sample of brown rat (*R. norvegicus*) and 150 samples of house mouse (*M. musculus*). Coronavirus RNA was only found in house mice (*M. musculus*) from El Hierro (10.53%), Tenerife (7.02%) and Lanzarote (5.26%) islands (Table 1). We found 11 sequences of the *Murine coronavirus* (M-CoV) (see accession numbers in Supplementary Material) belonging to the genus *Betacoronavirus* and subgenus *Embecovirus* (5). There were no significant differences in M-CoV prevalence between islands (Chi-square = 0.49, df = 2, *p* = 0.78). In addition, the prevalence of coronavirus found was similar along the years, 0.03% (3/109) in 2016, 0.06% (1/16) in 2017 and 2019 (2/33) and 0.05% in 2018 (5/102).

**Table 1:** Prevalence of coronavirus in rodents belonging to *Mus musculus, Rattus rattus* and *Rattus norvegicus* species from Canary Islands (Spain).

**Island**	**Location**	**Species**	**X/Y**	**P**
El Hierro	Frontera^*^	*Mus musculus*	2/19	10.53
		*Rattus rattus*	0/14	0.00
	**Total**	***Mus musculus***	**2/19**	**10.53**
		***Rattus rattus***	**0/14**	**0.00**
La Palma	Barlovento	*Rattus rattus*	0/1	0.00
	Breña Alta^*^	*Mus musculus*	0/2	0.00
		*Rattus rattus*	0/3	0.00
	El Paso	*Rattus rattus*	0/2	0.00
	Puntallana	*Mus musculus*	0/1	0.00
		*Rattus rattus*	0/1	0.00
	San Andrés y Sauces	*Rattus rattus*	0/4	0.00
	**Total**	***Mus musculus***	**0/3**	**0.00**
		***Rattus rattus***	**0/11**	**0.00**
Tenerife	Arona^*^	*Mus musculus*	0/1	0.00
		*Rattus rattus*	0/1	0.00
	El Batan	*Rattus rattus*	0/11	0.00
	El Sauzal^*^	*Rattus rattus*	0/1	0.00
	Fasnia	*Mus musculus*	0/1	0.00
		*Rattus rattus*	0/20	0.00
		*Rattus norvegicus*	0/1	0.00
	Guía de Isora	*Rattus rattus*	0/1	0.00
	La Guancha^*^	*Mus musculus*	0/2	0.00
		*Rattus rattus*	0/4	0.00
	La Laguna^*^	*Mus musculus*	2/19	10.53
		*Rattus rattus*	0/9	0.00
	La Orotava	*Mus musculus*	0/4	0.00
		*Rattus rattus*	0/8	0.00
	San Miguel Abona^*^	*Mus musculus*	1/1	100
	Santa Cruz^*^	*Mus musculus*	1/17	5.88
		*Rattus rattus*	0/7	0.00
	Tegueste^*^	*Rattus rattus*	0/1	0.00
	**Total**	***Mus musculus***	**4/57**	**7.02**
		***Rattus rattus***	**0/61**	**0.00**
		***Rattus norvegicus***	**0/1**	**0.00**
Lanzarote	Femés	*Mus musculus*	0/43	0.00
		*Rattus rattus*	0/1	0.00
	Haría^*^	*Mus musculus*	5/39	12.82
		*Rattus rattus*	0/3	0.00
	Maguez^*^	*Mus musculus*	0/1	0.00
		*Rattus rattus*	0/8	0.00
	**Total**	***Mus musculus***	**5/83**	**5.26**
		***Rattus rattus***	**0/12**	**0.00**
Canary Islands		***Mus musculus***	**11/150**	**7.33**
		***Rattus rattus***	**0/109**	**0.00**
		***Rattus norvegicus***	**0/1**	**0.00**

*X/Y, Number of positive individuals/number of analyzed individuals. P, M-CoV prevalence (%)*.

* *anthropic locality. Bold values summarize the total results obtained separately and together in the different islands studied*.

All CoV-positive house mice (*M. musculus*) were captured in urban environments. All house rats (*R. rattus*) and brown rats (*R. norvegicus*) were negative for CoV.

**Figure 2:**
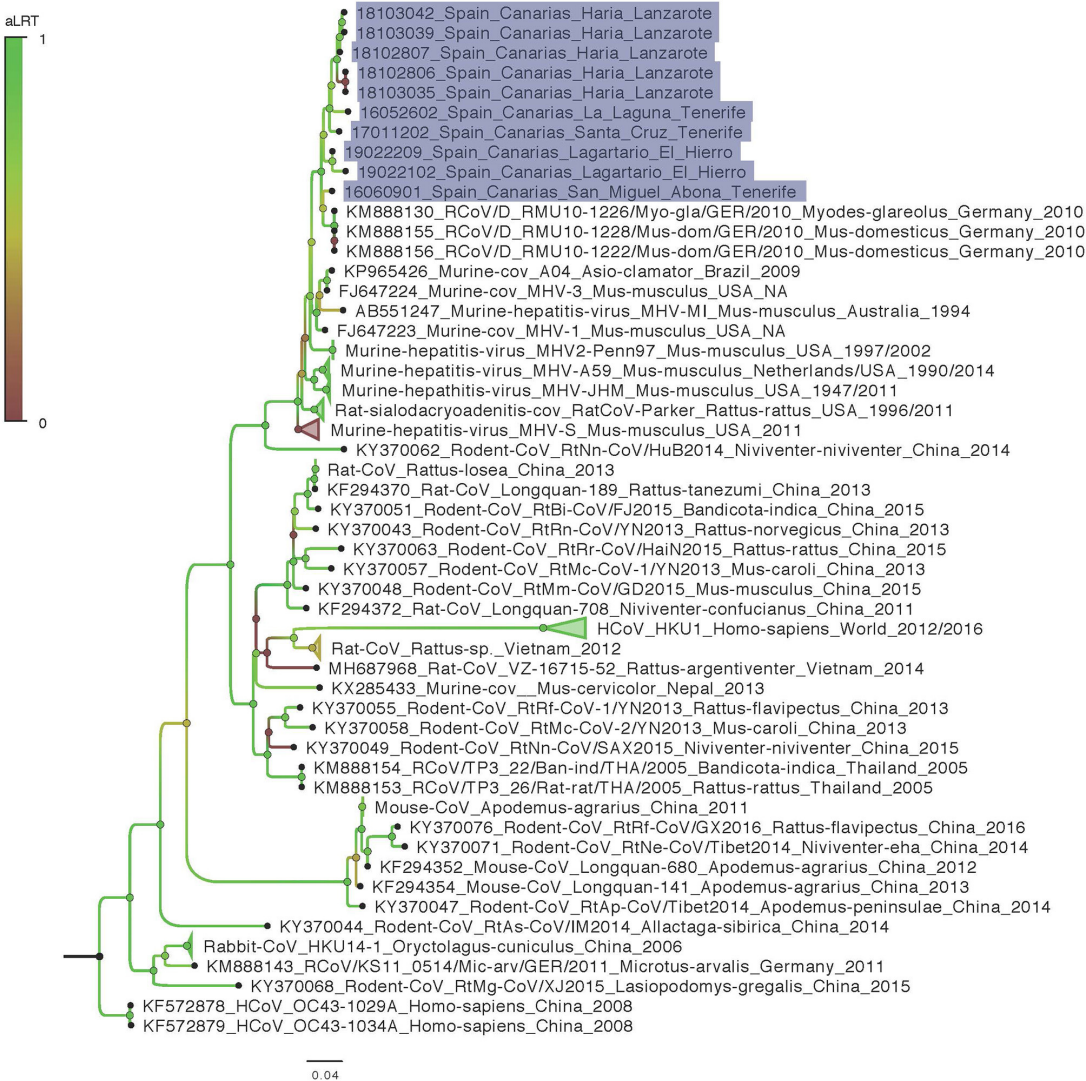
Maximum likelihood phylogeny of genetic sequences detected in the study with sequences representing the world diversity of *Coronavirinae Betacoronavirus Embecovirus*. Samples from this work are highlighted in purple. Names are built as follows for the reference sequences: Accession number_denomination_strain_host genus-species_country_date and for samples from this work that all originate from *Mus musculus* species: Isolate code_Locality_Island (NA, not available).

**Figure 3:**
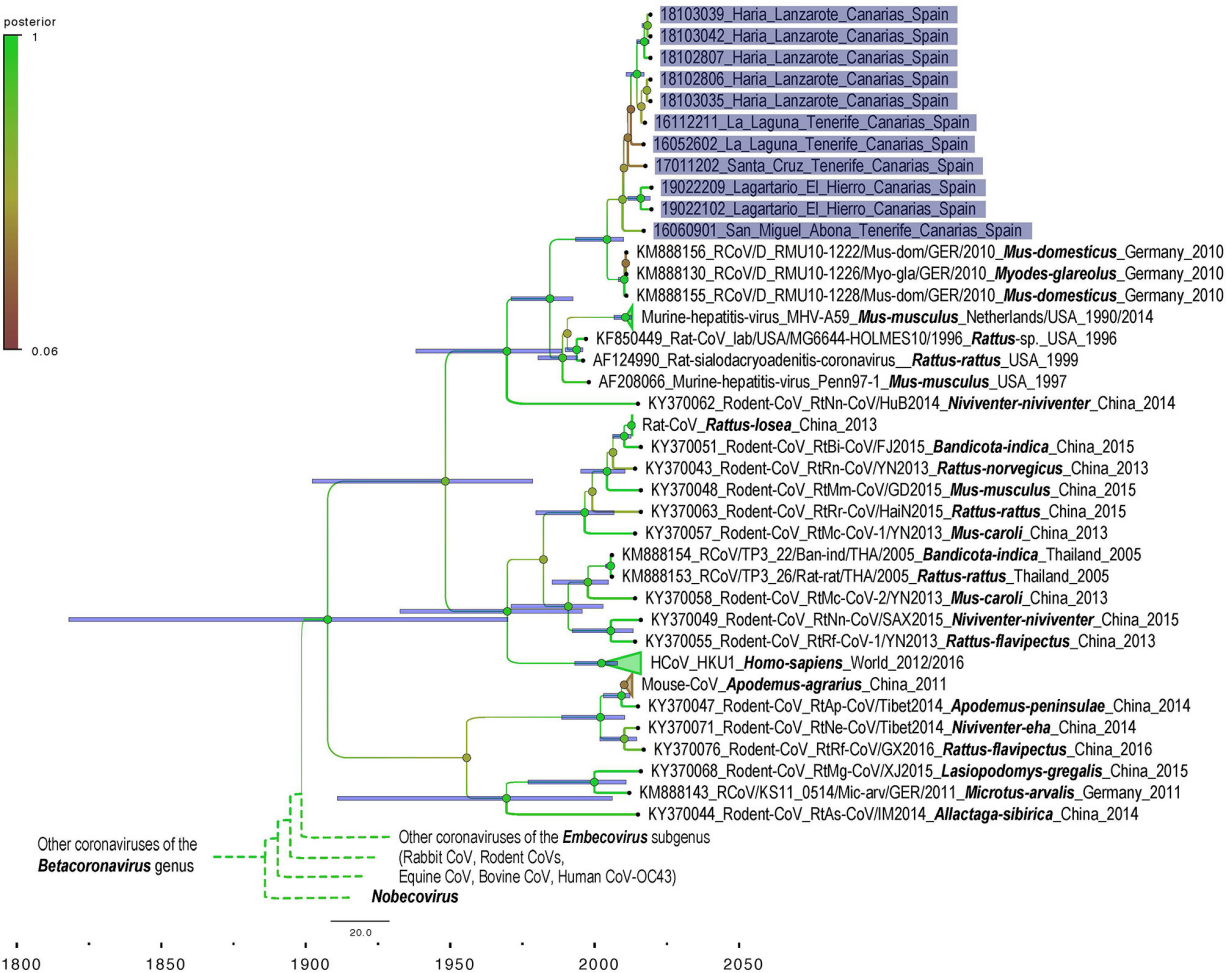
Bayesian phylogeny of genetic sequences representative of coronavirus strains detected in the study with sequences representing the world diversity of *Coronavirinae* subsampled from the dataset used for maximum likelihood phylogeny. Samples from this work are highlighted in purple. Names are built as for the Figure 2. Blue bars indicate 95% HPD interval around the mean value for each node.

We obtained 440 nuc by Sanger sequencing for most of our positive samples and therefore used a matrix cropped to 400 nucleotides for the phylogenetic analyses. Unfortunately, three samples failed to amplify 440 nuc despite several attempt and for these 3, only 190 nuc were included in the phylogenetic analyses. No difference in topology tree was observed when using more/less data in the phylogenetic analyses which indicate that these taxa were correctly placed in the tree despite the 3 shortest sequences. Moreover, these taxa (18102806 Haría Lanzarote; 18103035 Haría Lanzarote and 19022209 Lagartario El Hierro) all branch at a coherent position when considering other and more complete sequences from other taxa that cluster together by sites (by island). The phylogenetic analysis shows that the M-CoV found in the Canary Islands are related to CoV detected in 2010 by Drexler et al. in mice and bank vole (*Myodes glareolus*) from Germany (KM888130, KM888155-156) (Figure 2). The differentiation between Canary Islands and continental viruses seems to be quite recent (Figure 3). The phylogenetic analysis shows different clades of M-CoV for El Hierro, Tenerife and Lanzarote (Figure 2).

## Discussion

We report the first work on coronaviruses in rodent populations from the Canary Islands. We analyzed three rodent species but only one, the house mouse (*M. musculus*), was found positive for coronavirus RNA. Although a significant prospective effort was done, no coronaviruses could be detected in the 109 house rats (*R. rattus*) analyzed. These results suggest little or no circulation of coronaviruses in the house rat (*R. rattus*) populations studied. The very low sample size of brown rat (*R. norvegicus*), similar to other previous studies in these islands [e.g., (24)], does not allow us to estimate the incidence of coronaviruses in this species. Further studies are needed to know if coronaviruses are circulating in brown rat populations (*R. norvegicus*) of the Canary Islands.

All CoV-positive mice carried RNA of *Murine coronavirus* (M-CoV). This virus was isolated for the first time in 1947 (25). It is a highly contagious virus, frequently observed in laboratory mice, which has been used as a model of CoV to study hepatitis and demyelinating diseases such as multiple sclerosis in humans (26). M-CoV is transmitted primarily via aerosol or direct contact. The virus can be transmitted for a period of 30 days after infection. Though often subclinical, it can cause hepatitis, encephalitis, nephritis and enterocolitis in mice (27). M-CoV uses the N-terminal domain of its spike protein to bind the carcinoembryonic antigen-related cell adhesion molecule 1 (CEACAM-1a) receptor on the host cell membrane. Works on M-CoV have been mostly conducted on laboratory mice. In contrast, very few studies have been conducted on wild populations but phylogenetics analyses of available data enlighten the very opportunistic behavior of M-CoV or more widely of other members of the *Embecovirus* subgenus that are very diversified in multiple rodent host species in China (Figures 2, 3). These species may be capable of infecting multiple rodent host species (e.g., *Mus* and *Myodes* in Germany) and their diversity should be further investigated to better describe the potential role of genome plasticity in host range and evolution in this group.

In our study, house mice show a wide distribution in El Hierro, Tenerife and Lanzarote islands. However, all CoV-positive mice were found only in synanthropic environments, near inhabited localities, with a similar prevalence of M-CoV in the three islands. Also, the study revealed a diversity of M-CoV circulating in house mice in the three islands (Figure 2). In addition, we found different M-CoV variants circulating at the same time in the same mice population. This characteristic may increase the probability of co-infections in mice with the possible recombination between CoV strains. Several coronaviruses are known to recombine frequently, and this molecular mechanism is a main driving-force in their evolution (6, 28, 29). Unfortunately, due to the unique and short size region used for detection in this study, it is not possible to determine if recombination events have occurred, and the interpretation of temporal phylogenetic analyses are limited. Nevertheless, this marquer is located in the RNA dependent RNA polymerase (RdRp) coding region, a relatively conserved locus providing an accurate phylogenetic signal for this diversified group.

The phylogenetic analyses show a recent introduction of the M-CoV in the Canary Islands (with a mean in the early 2000's) and that the most prevalent of M-CoV Canary strains show higher similarity to the strains found by Drexler et al. (2) in house mice (*Mus musculus*) and bank voles (*Myodes glareolus*) in Germany (Figure 2). However, it should be considered that the sequences obtained in our study are short and additional data would enhance precision of the phylogenetic reconstruction. Although data on M-CoV is still scarce in the region, our data suggests that an European continental origin of the Canary Island virus, especially considering the close maritime relationships between Canary Islands and European countries.

Our globalized and connected human society contributes to the rapid spread of infectious agents. The world trade and population mobility increase fast and facilitate new connections between different naïve populations of hosts with new infectious agents. These changes are unprecedented in human history and represent a critical epidemiological factor that increases the risk of pathogens propagation. In this context, the spread of coronaviruses is a good example. Our work provides data suggesting a relatively recent introduction of M-CoV in the Canary Archipelago. In this sense, it reminds the importance of monitoring and controlling the arrival of species that are potential reservoirs of pathogens not only in the Canary Islands but also in all insular environments. On the other hand, the results obtained recently by Montagutelli et al. (30) show that the new COVID-19 strains (variants of concern, VOCs) can infect common laboratory mice, and replicate to high titers in the mouse lungs. These results reinforce the need for surveillance, intervention, and management strategies (31, 32).

## Data Availability Statement

The original contributions presented in the study are included in the article/Supplementary Material, further inquiries can be directed to the corresponding author/s.

## Ethics Statement

The animal study was reviewed and approved by Animal procedures were conducted in accordance with the Government Laws of 42/2007, 151/2001 (expedient numbers A/EST-030/2016 and AFF115/16) and RD630/2013 (expedient references EEI-007/2019, MRR/rsh and ADL/mjb).

## Author Contributions

JS-C, NM-C, PF, CF, and JM: conceived, designed the project and performed fieldwork. MA, ML-R, and AM: performed the analysis. All authors contributed to the article and approved the submitted version.

## Conflict of Interest

The authors declare that the research was conducted in the absence of any commercial or financial relationships that could be construed as a potential conflict of interest.

## Publisher's Note

All claims expressed in this article are solely those of the authors and do not necessarily represent those of their affiliated organizations, or those of the publisher, the editors and the reviewers. Any product that may be evaluated in this article, or claim that may be made by its manufacturer, is not guaranteed or endorsed by the publisher.
