# Supplementary material for: First Coronavirus Active Survey in Rodents From the Canary Islands
Source: Front Vet Sci. 2021 Aug 18;8:708079. doi: 10.3389/fvets.2021.708079 (PMC8416442; doi:10.3389/fvets.2021.708079)
Supplement: Supplementary file 1 [file Table_1.DOCX]

**Supplementary material:** accession numbers in GenBank of M-CoV from Canary Islands

| **Denomination strain and locality** | **Accession number** |
| --- | --- |
| 18103039_Spain_Canarias_Haría_Lanzarote | MZ061578 |
| 18103042_Spain_Canarias_Haría_Lanzarote | MZ061579 |
| 19022102_Spain_Canarias_Lagartario_El_Hierro | MZ061580 |
| 16112211_Spain_Canarias_La_Laguna_Tenerife | MZ061581 |
| 17011202_Spain_Canarias_Santa_Cruz_Tenerife | MZ061582 |
| 18102807_Spain_Canarias_Haría_Lanzarote | MZ061583 |
| 16060901_Spain_Canarias_San_Miguel de Abona_Tenerife | MZ061584 |
| 16052602_Spain_Canarias_La_Laguna_Tenerife | MZ061585 |
| 18103035_Spain_Canarias_Haría_Lanzarote | MZ061586 |
| 19022209_Spain_Canarias_Lagartario_El_Hierro | MZ061587 |
| 18102806_Spain_Canarias_Haría_Lanzarote | MZ061588 |
